# Nonlinear Effects of Linearly Increasing Perceptual Load on ERPs to Emotional Pictures

**DOI:** 10.1093/texcom/tgaa040

**Published:** 2020-07-29

**Authors:** Sebastian Schindler, Laura Gutewort, Maximilian Bruchmann, Robert Moeck, Thomas Straube

**Affiliations:** Institute of Medical Psychology and Systems Neuroscience, University of Muenster, Münster D-48149, Germany; Otto Creutzfeldt Center for Cognitive and Behavioral Neuroscience, University of Muenster, Münster D-48149, Germany; Institute of Medical Psychology and Systems Neuroscience, University of Muenster, Münster D-48149, Germany; Institute of Medical Psychology and Systems Neuroscience, University of Muenster, Münster D-48149, Germany; Otto Creutzfeldt Center for Cognitive and Behavioral Neuroscience, University of Muenster, Münster D-48149, Germany; Institute of Medical Psychology and Systems Neuroscience, University of Muenster, Münster D-48149, Germany; Institute of Medical Psychology and Systems Neuroscience, University of Muenster, Münster D-48149, Germany; Otto Creutzfeldt Center for Cognitive and Behavioral Neuroscience, University of Muenster, Münster D-48149, Germany

**Keywords:** IAPS EEG/ERP, load by emotion interaction, perceptual competition, perceptual load, processing resources

## Abstract

The prioritized processing of emotional as compared to neutral stimuli is reflected in enlarged event-related potentials (ERPs). However, perceptual load theory proposes that under conditions of high perceptual load, information processing is attenuated or abolished. The parametrical effects of load on ERPs to emotional pictures are unknown. To shed light on this question, the current preregistered ERP study (*N* = 30) systematically investigated the effects of load on ERPs to task-irrelevant negative, neutral, and positive pictures. Crucially, while perceptual input was held constant, perceptual load was systematically manipulated so that it increased linearly across 4 load levels, which was evident in behavioral data. In contrast, load effects on ERP differences between emotional and neutral stimuli did not follow a linear function. For the N1, early posterior negativity and late positive potential, a nonlinear function with reversed emotion effects at the third load level provided the best fit. These findings do not only show that perceptual load attenuates emotional picture processing but also suggest that active processes are initiated to reduce distraction by emotional information. Moreover, these effects of perceptual load on emotional ERP components appear to deviate from theoretically expected functions.

## Introduction

Event-related potentials (ERPs) show a prioritized processing of emotional stimuli, which is based on their biological relevance (e.g., motivated attention, see Lang et al. 1997; Schupp, Öhman, et al. 2004b; Schupp et al. 2006). Emotional pictures therefore lead to distinct and pronounced ERP modulations, typically reflected in an enlarged early posterior negativity (EPN) and late positive potential (LPP). The EPN component peaks between 200 and 400 ms after stimulus onset over occipito-temporal regions and indicates early attention mechanisms (e.g., Schupp, Öhman, et al. 2004b). The LPP is a component that arises approximately 400 ms after stimulus onset and is associated with stimulus evaluation and controlled attention processes (e.g., Hajcak et al. 2009). Also, early ERP components (P1, N1) reflecting earlier stages of stimulus detection and discrimination (e.g., Hillyard and Anllo-Vento 1998; Herrmann and Knight 2001) might be differentially affected by the emotional content (see Olofsson et al. 2008).

Even though emotional information is prioritized, the question arises if, and if so, how such an increased processing of emotional pictures depends on available attentional resources. For example, perceptual load theory proposes reduced processing of distracter stimuli under high perceptual load, which is supposed to attenuate or abolish distracter processing at early stages (Lavie and Tsal 1994; Lavie et al. 2004, 2014). While there are no absolute viewpoints, some theoretical accounts suggest that emotional information is at least to a certain extent immune to attentional manipulations (for a review, see Vuilleumier and Huang 2009; specifically regarding behavioral effects, see Carretié 2014), and others propose that the processing of emotional stimuli strongly depends on available resources (e.g., see Pessoa 2009; Pessoa et al. 2013). Previous studies have provided mixed findings regarding perceptual load effects on ERPs to emotional pictures, with studies reporting reduced EPN effects (Schupp, Stockburger, Bublatzky, et al. 2007a; Schupp et al. 2008) and other studies finding no load effects on an emotional EPN and LPP differentiation (Norberg et al. 2010; Sand and Wiens 2011; Wiens et al. 2012). In addition to these inconsistent results, 2 major problems can be identified when examining previous research on perceptual load effects: Firstly, there is lack of definition that could clarify what represents high and what low perceptual load (Murphy et al. 2016). Secondly, high perceptual load is typically achieved by increasing the number of distracters or the set-size. However, this leads to perceptual confounds that make it difficult to examine and interpret the ERP effects of interest (e.g., size, contrast, eccentricity, see Hughes 1984; Johannes et al. 1995; Wijers et al. 1997; Busch et al. 2004). Thus, it is challenging but highly important to control the perceptual input while at the same time manipulate perceptual task difficulty.

To resolve the question of how perceptual load affects emotional ERP modulations, a range of linearly increasing perceptual load levels should be realized, while perceptual input needs to be kept constant. The use of multiple load levels can also shed light on the exact parametric load-emotion ERP interaction that could take the form of a linear, a quadratic, or even a cubic function. While linearly increasing perceptual load should theoretically decrease emotional ERP effects in a linear fashion, finding non-linear functions would be highly informative as well: Such functions would indicate that study designs with only 2—or even 3—load levels might lead to the wrong conclusions. Such studies would miss information about reduced emotion effects depending on a specific load level. For example, quadratic trends would indicate a vertex function, suggesting lowest or highest effects for intermediate load levels. The current preregistered study investigated how ERPs to emotional pictures depend on perceptual load by using a novel perceptual load task with 4 load levels but constant perceptual input. In particular, we explored whether a linear increase in perceptual load leads to linear effects on early, mid-latency and late ERP components to emotional versus neutral pictures.

## Materials and Methods

### Participants

After piloting the design, an initial sample of 31 participants was tested. One participant had to be excluded as after testing, s/he reported to have suffered from a systemic connective disorder in the past. The final sample consisted of 30 participants (23 female) who were on average 23.47 years old (SD = 2.85). All participants had normal or corrected-to-normal vision, were right-handed and had no reported history of neurological or psychiatric disorders. All participants gave written informed consent and received 10 euros per hour for participation. To make sure that they would prioritize the load task, they received a performance-dependent bonus for the load task of up to 7.20 euros (rewarding correct responses, see below).

### Stimuli

A set of 60 negative, 60 neutral and 60 positive pictures (Steppacher et al. 2015) was taken from the IAPS system (Lang et al. 2008). The pictures were rated in terms of valence (1 = highly negative, 7 = highly positive; *M*_valence negative_ = 2.45, SD = 0.42, *M*_valence neutral_ = 5.06, SD = 0.28, *M*_valence positive_ = 7.33, SD = 0.75) and arousal (1 = not arousing, 7 = highly arousing; *M*_arousal negative_ = 5.82, SD = 0.79, *M*_arousal neutral_ = 3.57, SD = 1.04, *M*_arousal positive_ = 5.67, SD = 0.74). Regarding the arousal ratings, an independent *t*-test showed no difference between negative and positive images (*t*_(1,118)_ = 1.08, *P* = 0.284; for effects of arousal, see e.g., Schubring and Schupp 2019). Moreover, image statistics were compared with respect to brightness and showed no significant differences between the image sets (*F*_(2,177)_ = 2.95, *P* = 0.055; Torralba and Oliva 2003; Bainbridge and Oliva 2015). To test for frequency differences, we firstly compared the relative power across frequencies between the emotion categories. Additionally, we calculated the relative power across spatial frequencies for each base color separately. Global and color-specific frequency power maps showed that confidence intervals overlapped between negative, neutral and positive images in lower frequencies (for details see the Supplementary Material). For higher spatial frequencies (>30 cycles per image), confidence intervals between positive and negative pictures did not overlap, thus indicating a higher power of positive pictures in higher spatial frequencies.

### Procedure

**Figure 1:**
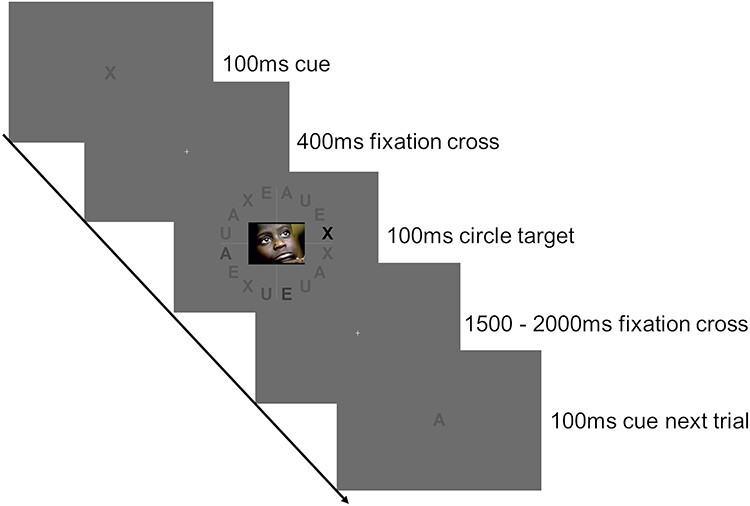
Illustration of the trial structure and the employed task. A respective letter is cued (in this case x), and the darkest of all x letters has to be detected. Three other letters darker than standard gray are displayed for the not cued letters a, u, and e, which were not task relevant.

Participants were seated 60 cm in front of a gamma-corrected display (Iiyama G-Master GB2488HSU) running at 60 Hz with a Michelson contrast of 0.9979 (*L*_min_ = 0.35 cd/m^2^; *L*_max_ = 327.43 cd/m^2^). The background was set to medium gray (RGB 109109109), corresponding to the average image luminance of 163.89 cd/m^2^. To manipulate perceptual load while keeping physical stimulus properties constant, we used a novel paradigm (see Fig. 1) consisting of 16 letters evenly spaced on a circle with a radius of 8.8°. Each letter was 1.6° wide and 2.2° high. The circle was divided into 4 quadrants by a horizontal and a vertical light gray line extending through the display center. Each quadrant contained 4 letters (A, X, E, and U) in a randomized order, one or maximal 2 being of darker font. The complete display thus contained 4 instances of each letter, 3 of which were presented at a ‘standard grey level’ (RGB 90, 90, 90), one being slightly or severely darker, depending on the load difficulty (see below and Fig. 1). The letters were chosen based on their nearly identical surface area when printed in bold Arial font.

Before the measurement began, participants were instructed to avoid eye movements and blinks during stimulus presentation. Each trial then started with the display of a cue, which informed participants about the letter they should attend to. Their task was to indicate the quadrant containing the one instance of the cued letter that was darker than the standard gray. Participants used 2 fingers of each hand to complete the 4 choice task (response keys: a, y, m, k on a keyboard with QWERTZ-layout). Participants were required to indicate if the darkest one of the cued letter (e.g., darkest X in the example Fig. 1) was located in the upper left quadrant (requiring them to press a), the lower left quadrant (press y), the upper right quadrant (press k), or the lower right quadrant (press m). The target letter was presented at one out of 4 gray levels (RGBs 0, 0, 0 (load level one, easiest); 60, 60, 60; 72, 72, 72; or 80, 80, 80 (load level 4, most difficult)), which had been chosen carefully based on intensive pilot experiments to ensure a linear increase in discrimination difficulty. Importantly, the display always contained 4 letters deviating from the standard gray, that is, one of each letter at one of the 4 gray levels. The assignment of gray levels to letters was randomized and counterbalanced across trials so that cueing the letter X, for example, was not indicative of its gray level. This procedure ensured the display of identical perceptual information while manipulating perceptual load. All 12 conditions (3 emotion levels: negative, neutral, positive, by 4 load levels: load1, load2, load3, load4) were presented in random order. Each condition consisted of 60 trials, summing up to a total of 720 trials.

During the experiment, load was manipulated trial-wise. Each trial began with the presentation of a letter cue for 100 ms, which was followed by a fixation cross displayed for 400 ms. After the cueing, the letter circle, containing 16 letters and an emotional or neutral picture distracter in the center, was presented for 100 ms. During the intertrial interval (ITI), a fixation cross was presented for 2000–2500 ms before the next trial started. Participants could rest during 6 self-paced breaks. In each break, participants were informed about their performance during the last block.

### E‌EG Recording and Preprocessing

EEG signals were recorded from 64 BioSemi active electrodes using Biosemi’s Actiview software (www.biosemi.com). Four additional electrodes measured horizontal and vertical eye movements. Recording sampling rate was 512 Hz. Offline data were re-referenced to average reference, and filtered with a high-pass forward filter of 0.01 (6 db/oct) as well as a 40-Hz low-pass zero phase filter (24 db/oct). Recorded eye movements were corrected using the automatic eye-artifact correction method implemented in BESA (Ille et al. 2002). Remaining artifacts were rejected based on absolute threshold (120 μV), gradient (75), and low signal change (0.01). Noisy electrodes were identified through visual inspection and interpolated using a spline interpolation procedure. Filtered data were segmented from 100 ms before stimulus onset to 1000 ms after stimulus presentation. Baseline correction used the 100 ms before stimulus onset. All participants fulfilled the preregistered inclusion criteria related to EEG/ERP data. On average, 2.1 (SD = 1.35) electrodes were interpolated, and 55 trials (~92%) were kept for averaging. There were no differences in kept trials between emotion (*F*_(2,58)_ = 0.65, *P* = 0.525, partial η^2^ = 0.022) or load conditions (*F*_(3,87)_ = 1.18, *P* = 0.324, partial η^2^ = 0.039), and no interaction of load and emotion (*F*_(4.47129.65)_ = 1.64, *P* = 0.161, partial η^2^ = 0.054).

### Behavioral and EEG Data Analyses

Behavioral data were analyzed using the program JASP (wwww.jasp.org). EEG scalp data were statistically analyzed and visualized using EMEGS. Three (Emotion: negative, neutral, positive) by 4 (Load: load1, load2, load3, load4) repeated measures analyses of variance (ANOVAs) were conducted to investigate main effects of perceptual load and emotion as well as their interaction for reaction time, response accuracy and ERP components of interest. For the EPN, laterality (left vs. right) was included as an additional factor. Partial eta-squared (partial η^2^) was used to describe effect sizes (Cohen 1988). For interaction effects of load and emotion, additional polynomial trends were calculated to compare linear, quadratic and cubic trends modeling the emotion effect as a function of perceptual load for differences between negative and neutral and between positive and neutral pictures. Time windows of interest were based on previous studies using this perceptual load paradigm (Schindler et al. 2019), and a high-powered study using the same pictures but without a concurrent load task (Schindler and Straube 2020). The P1 was measured from 80 to 100 ms, the N1 from 110 to 170 ms, the EPN from 280 to 380 ms, and the LPP from 400 to 700 ms. We used occipital sensor clusters for the P1 (O1, Oz, O2), the N1 (Oz, Iz, O1, P9, PO7, O2, P10, PO8) and the EPN component (left O1, P9, P7, PO7; right O2, P10, P8, PO8). For the LPP, a centro-parietal cluster was used (C3, C1, Cz, C2, C4, CP3, CP1, CPz, CP2, CP4). Since we were interested in perceptual load difficulty modulatory effects on differences between emotional and neutral pictures, we included all trials for our main analyses. However, to exclude that our observed effects are selectively affected by induced error processing, we additionally calculated the same ERP analyses for correct trials only (see the Supplementary Materials Section 3). Furthermore, to account for differences in eye-related activity, we performed analyses on horizontal and vertical eye movements measured by the EOG channels. The detailed registration and all data can be retrieved from the OSF platform (https://osf.io/8rs7b/), which is linked to preregistration (https://osf.io/qn38r).

## Results

### Behavior

**Figure 2:**
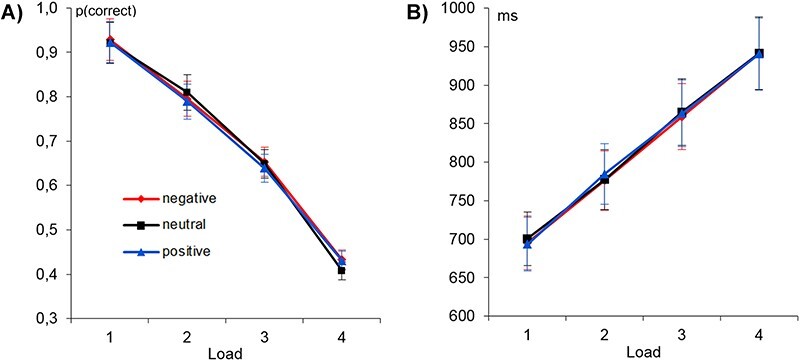
Accuracy and reaction time across all load levels and emotions. (*a*) Accuracy (percent correct) and (*b*) Reaction time in ms. Error bars depict the 95% confidence interval of the mean.

For response accuracy, no main effect of emotion (*F*_(2,58)_ = 1.06, *P* = 0.352, partial η^2^ = 0.035), but a main effect of load was detected (*F*_(1.49,43.31)_ = 210.70, *P <* 0.001, partial η^2^ = 0.879; see Fig. 2). With respect to the main load effect, polynomial trends showed that accuracy values decreased linearly with increasing load (linear trend: *F*_(1,29)_ = 273.07, *P <* 0.001; partial η^2^ = 0.904; explained 99% of the variance; quadratic: *F*_(1,29)_ = 13.39, *P* = 0.001; partial η^2^ = 0.316; 1% variance explained; cubic: *F*_(1,29)_ = 0.79, *P* = 0.382; partial η^2^ = 0.026; 0% variance explained). There was no significant interaction of emotion and load (*F*_(3.73108.07)_ = 0.15, *P* = 0.956, partial η^2^ = 0.005). For reaction time, no main effect of emotion (*F*_(2,58)_ = 0.57, *P* = 0.567, partial η^2^ = 0.019), but a main effect of load was detected (*F*_(3,87)_ = 689.08, *P <* 0.001, partial η^2^ = 0.960; see Fig. 2). Polynomial trends again showed linearly decreasing reaction times with increasing load (linear: *F*_(1,29)_ = 1358.35, *P <* 0.001; partial η^2^ = 0.979; explained 98% of the variance; quadratic: *F*_(1,29)_ = 38.32, *P <* 0.001; partial η^2^ = 0.569; 2% variance explained; cubic: *F*_(1,29)_ = 2.77, *P* = 0.107; partial η^2^ = 0.087; 0% variance explained). Again, there was no significant interaction of emotion and load (*F*_(6,174)_ = 1.07, *P* = 0.386, partial η^2^ = 0.035).

### ERPs

#### P1

**Figure 3:**
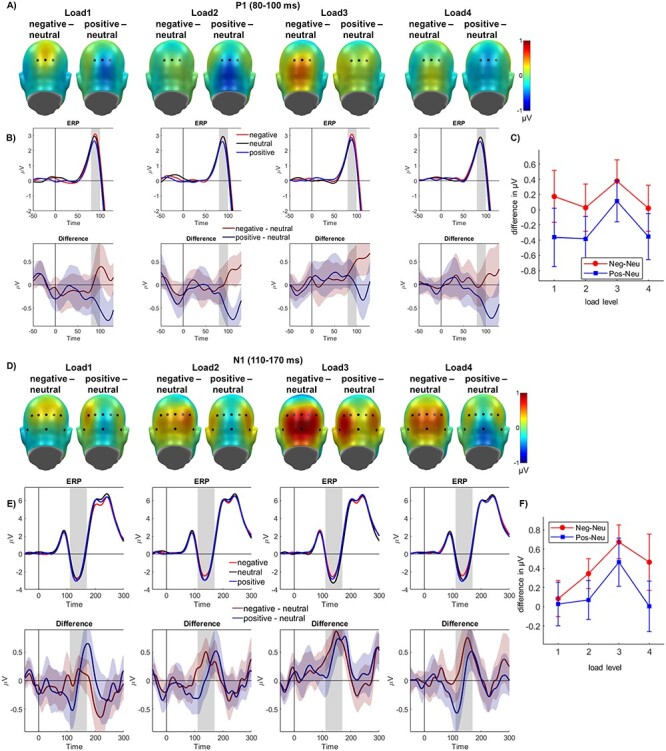
P1 (*A*–*C*) and N1 (*D*–*F*) effects. (*A* and *D*) Difference topographies between negative and neutral and between positive and neutral pictures for each load level, highlighting the electrodes for the P1 (A) and N1 (D) ROI. (*B* and *E*) Averages for all emotion conditions over the P1 (*B*) and N1 (*E*) electrode clusters and their difference plots (negative-neutral and positive-neutral), displayed separately for each load level. (*C* and *F*) Average of differences (negative-neutral and positive-neutral) over the entire P1 (*C*) and N1 (*F*) time window for all load levels. Error bars depict 95% CIs.

Regarding the P1, a main effect of emotion was found (*F*_(2,58)_ = 6.83, *P* = 0.002, partial η^2^ = 0.191; see Fig. 3*A*–*C*), but no main effect of perceptual load was detected (*F*_(3,87)_ = 0.36, *P* = 0.784, partial η^2^ = 0.012). With respect to the main emotion effect, negative pictures elicited a larger P1 amplitude when compared to positive (*P* = 0.002), but not when compared to neutral pictures (*P* = 0.165). P1 amplitudes elicited by neutral pictures fell in between the negative and positive emotion conditions, with significantly larger values than in the positive condition (*P* = 0.026). There was no significant interaction of emotion and load (*F*_(6,174)_ = 0.95, *P* = 0.462, partial η^2^ = 0.032).

**Figure 4:**
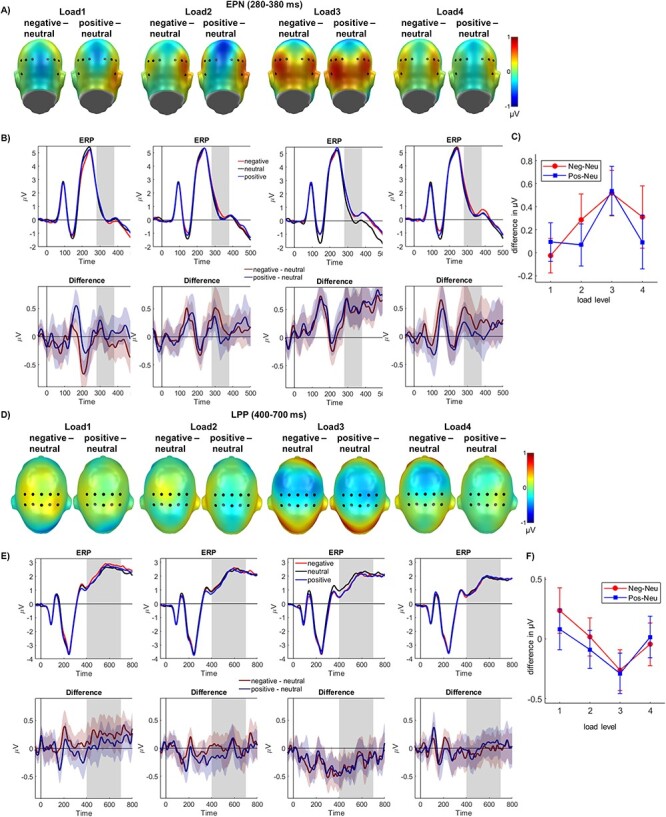
EPN (*A*–*C*) and LPP (*D*–*F*) effects. (*A* and *D*) Difference topographies between negative and neutral and between positive and neutral pictures for each load level, highlighting the electrodes for the EPN (*A*) and LPP (*C*) ROI. (*B* and *E*) Averages for all emotion conditions over the P1 (*B*) and N1 (*E*) electrode clusters and their difference plots (negative-neutral and positive-neutral), displayed separately for each load level. (*C* and *F*) Average of differences (negative-neutral and positive-neutral) over the entire EPN (*C*) and LPP (*F*) time window for all load levels. Error bars depict 95% CIs.

**Figure 5:**
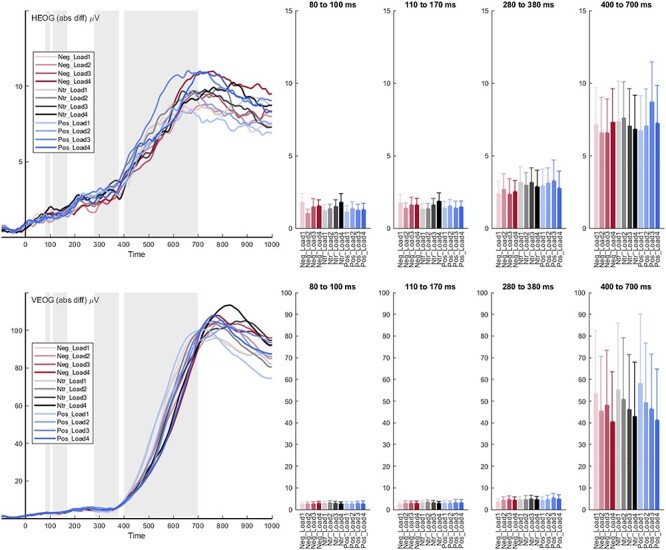
Eye activity across all emotion and load conditions. The upper bar depicts average horizontal (HEOG) mean activity per time window, the lower vertical eye activity (VEOG). Red color indicates negative, black neutral, and blue positive conditions, with darker font for higher load levels.

#### N1

For the N1, a main effect of emotion (*F*_(2,58)_ = 15.77, *P <* 0.001, partial η^2^ = 0.352), but no main effect of perceptual load was found (*F*_(3,87)_ = 0.61, *P* = 0.608, partial η^2^ = 0.021). Regarding the main effect of emotion, negative pictures elicited a smaller N1 amplitude compared to both neutral (*P <* 0.001) and positive pictures (*P* = 0.002). There was no significant difference between N1 amplitudes elicited by neutral and positive pictures (*P* = 0.125). Importantly, there was a significant interaction of emotion and load (*F*_(6,174)_ = 2.18, *P* = 0.048, partial η^2^ = 0.070; see Fig. 3*D*–*F*). Polynomial trends were computed for amplitude differences between negative and neutral pictures, showing no significant linear (*F*_(1,29)_ = 3.56, *P* = 0.069, partial η^2^ = 0.109; 55% variance explained), quadratic (*F*_(1,29)_ = 4.15, *P* = 0.051, partial η^2^ = 0.125; 35% variance explained) or cubic trend (*F*_(1,29)_ = 1.19, *P* = 0.284, partial η^2^ = 0.040; 10% variance explained). When examining such differences between positive and neutral pictures, there were no significant linear (*F*_(1,29)_ = 0.12, *P* = 0.732, partial η^2^ = 0.044; 2% variance explained) or quadratic contrasts either (*F*_(1,29)_ = 3.75, *P* = 0.062, partial η^2^ = 0.115; 50% variance explained). However, a cubic trend reached significance (*F*_(1,29)_ = 6.57, *P* = 0.016, partial η^2^ = 0.185; 48% variance explained).

#### EPN

With respect to the EPN, both main effects of emotion (*F*_(2,58)_ = 7.19, *P* = 0.002, partial η^2^ = 0.199;) and perceptual load reached significance (*F*_(3,87)_ = 10.14, *P <* 0.001, partial η^2^ = 0.259). Regarding the main effect of emotion, neutral pictures elicited a larger EPN than negative (*P* = 0.003) and positive stimuli (*P* = 0.007). The latter negative and positive conditions did not exhibit significantly different amplitude values (*P* = 0.275). For the main effect of load, polynomial trends showed a linear decrease in EPN amplitude values with increasing load (*F*_(1,29)_ = 23.61, *P <* 0.001, partial η^2^ = 0.449; 87% variance explained).

In addition, there was a significant interaction effect of emotion and load (*F*_(6,174)_ = 2.87, *P* = 0.011, partial η^2^ = 0.090; see Fig. 4*A*–*C*). Here, polynomial trends computed for differences between negative and neutral pictures showed a significant linear (*F*_(1,29)_ = 5.86, *P* = 0.022, partial η^2^ = 0.168; 50% variance explained) and a significant quadratic trend (*F*_(1,29)_ = 4.572, *P* = 0.041, partial η^2^ = 0.136; 44% variance explained), while a cubic contrast did not reach significance (*F*_(1,29)_ = 0.53, *P* = 0.473, partial η^2^ = 0.004; 6% variance explained). Regarding the differences between positive and neutral pictures, there was no significant linear (*F*_(1,29)_ = 0.86, *P* = 0.363, partial η^2^ = 0.116; 8% variance explained) or quadratic trend (*F*_(1,29)_ = 3.13, *P* = 0.088, partial η^2^ = 0.097; 28% variance explained), but a cubic contrast reached significance (*F*_(1,29)_ = 6.57, *P* = 0.016, partial η^2^ = 0.185; 64% variance explained).

#### LPP

Regarding the LPP, no significant main effect of emotion was found (*F*_(2,58)_ = 1.07, *P* = 0.351, partial η^2^ = 0.035), but a main effect of perceptual load was detected (*F*_(1.80,52.09)_ = 23.39, *P <* 0.001, partial η^2^ = 0.446). Polynomial trends showed a significant linear trend of decreasing LPP amplitudes with increasing load (*F*_(1,29)_ = 32.82, *P <* 0.001, partial η^2^ = 0.531; 99% variance explained).

Furthermore, there was a significant interaction of emotion and load (*F*_(6,174)_ = 2.46, *P* = 0.026, partial η^2^ = 0.078; see Fig. 4*D*–*F*). Polynomial trends computed for the differences between negative and neutral pictures showed no significant linear (*F*_(1,29)_ = 4.12, *P* = 0.052, partial η^2^ = 0.124; 52% variance explained), or cubic contrast (*F*_(1,29)_ = 1.55, *P* = 0.223, partial η^2^ = 0.051; 12% variance explained), but a significant quadratic trend (*F*_(1,29)_ = 4.52, *P* = 0.042, partial η^2^ = 0.135; 36% variance explained). With regard to the differences between positive and neutral pictures, there was no significant linear (*F*_(1,29)_ = 0.92, *P* = 0.345, partial η^2^ = 0.345; 11% variance explained), or cubic trend (*F*_(1,29)_ = 1.14, *P* = 0.295, partial η^2^ = 0.038; 14% variance explained), but a significant quadratic trend (*F*_(1,29)_ = 7.27, *P* = 0.012, partial η^2^ = 0.201; 75% variance explained).

#### Control Analyses of Horizontal and Vertical Eye Activity

For horizontal eye movements, we did not observe main effects of load for any of the investigated ERP components (*Fs*_(3,87)_ < 1.37 *Ps* > 0.255; see Fig. 5). There were also no main effects of emotion (*Fs*_(2,58)_ < 2.40 *Ps* > 0.099), and no interaction effects between load and emotion for the N1, EPN, and LPP (*Fs*_(6174)_ < 1.76 *Ps* > 0.110). Regarding vertical eye movements, there were no main effects of load for the P1, N1, and EPN (*Fs*_(3,87)_ < 1.15 *Ps* > 0.332), but a significant effect for the LPP (*F*_(3,87)_ = 5.00 *P* = 0.003). Here, larger vertical activity in the LPP window was found for lower load levels, across all emotion conditions (see Fig. 5). There were no main effects of emotion (*Fs*_(2,58)_ < 0.92 *Ps* > 0.405), and no interactions for all ERPs (*Fs*_(6174)_ < 0.83 *Ps* > 0.551).

## Discussion

The goal of the present study was to examine how the neural processing of task-irrelevant emotional and neutral pictures is affected by linearly increasing perceptual load. We observed no main effect of load and no load by emotion interaction effect for the P1. The effect of perceptual load on emotion processing started with the N1, encompassed the EPN and lasted until the LPP time window. At early processing stages (N1 and EPN), negative emotion effects were explained similarly well by linear and quadratic functions of perceptual load. For positive-neutral differences, quadratic and cubic contrasts explained large proportions of N1 and EPN variance. At late processing stages, emotion differences systematically followed a quadratic shape. Nevertheless, a consistent finding across all components pertains to the maximal load effect on emotional processing on the intermediate, third load level. Finally, main effects of load were found for the EPN and LPP—here, an increase in perceptual load led to linearly decreasing EPN and LPP amplitudes.

The earliest ERP component investigated in this study was the P1. Here, we observed neither a significant main effect of load nor a significant load by emotion interaction, even though the overall pattern of amplitude values across conditions was descriptively similar to the subsequently investigated components. This might be explained by the notorious unreliability of P1 effects (for its grand average reliability, see Gaspar et al. 2011), and the associated differences in low-level features (see Schindler et al. 2019; for a more detailed discussion of limiting aspects regarding the found P1 main effect of emotion, see the Supplementary Material).

For the subsequent N1, we found no main effect of perceptual load either, but, in contrast to the P1, a significant load by emotion interaction. Here, no emotional differentiation was detected on the lowest level of perceptual load. At higher levels of perceptual load, an amplitude decrease for positive, but especially for negative emotional pictures was found (see Fig. 4*C*). This interaction suggests that emotion effects are not simply absent but that reversed emotion effects are found at higher load levels (in our study from load levels 2–4, with a maximal effect at level 3). These effects on the N1, a component which is related to early stimulus amplification (Neville and Lawson 1987; Eimer 1994; Marzecová et al. 2018), call for a more thorough examination: The decrease in and even reversal of emotion processing cannot be explained by a simple resource account such as Lavie’s perceptual load theory (2014). While perceptual load does indeed modulate early, sensory-related components, the reversed effects identified in our study suggest some additional inhibition processes.

A repetition of this pattern was found at later stages, showing similar reversed emotion effects on the EPN, with no or only slight initial emotion effects at the first load level followed by decreased emotion effects at higher load levels. However, in the absence of such load manipulations, pronounced negativities are consistently observed for emotional compared to neutral pictures (e.g., see Junghöfer et al. 2001; Schupp et al. 2006; Steppacher et al. 2015). Moreover, the very same IAPS images have been validated to elicit a pronounced EPN under conditions without perceptual competition (Schindler and Straube 2020). While our findings are in contrast to studies showing no effects of perceptual load on the EPN and LPP (Sand and Wiens 2011; Wiens et al. 2012), they are in line with the reported reduction of emotion effects during a concurrent competitive perceptual or auditory tasks (e.g., see Schupp, Stockburger, Bublatzky, et al. 2007a; Schupp et al. 2008). This empirical discrepancy might partly be explained by the employed load manipulation: Perceptual load seems to have a stronger interference effect when it occurs in the periphery, which was the case in our study (for differences between effects of central and peripheral load on driving performance, see Marciano and Yeshurun 2015). As the EPN is related to early attentional selection processes (Schupp, Junghöfer, et al. 2004a; Wieser et al. 2010), our EPN results suggest active mechanisms that minimize distracting influences from emotional stimuli to maintain high performance levels in the task. More specifically, the results of our study imply that participants actively shielded their available attentional resources, which was reflected in decreased EPN amplitudes towards all distracters, but especially towards emotional stimuli. Given the main and interaction effects, the EPN might represent a “bottleneck” in elaborate emotion processing, where attention competes with emotional differentiation (e.g., see Schupp, Stockburger, Bublatzky, et al. 2007a; Schindler et al. 2020). For the very same perceptual load paradigm, a similar pattern of reversed EPN effects has been observed for emotional faces (Schindler et al. 2019, Supplementary Material).

For the LPP, similar interactions of load and emotion were found. The LPP is related to the controlled evaluation of emotionally relevant stimuli (Schupp et al. 2006; Hajcak et al. 2009). Emotional amplifications at this stage are strongly influenced by the focus of the task, such as task relevance and emotional appraisal (Moser et al. 2006; Schupp, Stockburger, Codispoti, et al. 2007b; Schindler and Kissler 2016; for a review, see Hajcak et al. 2010). This also implies that processes at this stage are highly vulnerable to competing perceptual tasks. In line with these factors affecting the LPP, studies manipulating feature-based attention have reliably shown that—in contrast to earlier stages—LPP emotion effects heavily depend on the attentional focus (Rellecke et al. 2012; Wiens et al. 2012).

Contradicting previous studies reporting perceptual load effect on the emotional LPP modulation (Norberg et al. 2010; Wiens et al. 2012; Wiens and Syrjänen 2013), our study provides strong evidence for emotion effects as a quadratic function of perceptual load, reaching its maximum on the third load level. As noted above, participants were motivated to prioritize the perceptual load task as they earned a performance-dependent bonus. Such rewards serve as goals of voluntary actions, leading to a maintenance of or an increase in the rewarded behavior (Schultz 2000).

The observed nonlinear ERP effects need to be incorporated into a broader theoretical framework. Theoretical accounts have already aimed to explain nonlinear perceptual load effects for increasing set sizes (Giesbrecht et al. 2014). Some recent theories explain such effects by neural competition processes (e.g., see Scalf et al. 2013; Giesbrecht et al. 2014). In the neural theory of visual attention, the authors propose that in a first forward process, all display items are processed, while they compete with each other in a second wave of selective processing (Giesbrecht et al. 2014). In a similar vein, competition rather than limited capacities is thought to hinder representation of stimuli (Scalf et al. 2013). Importantly, here top-down filtering not only enhances target representation but suppresses other irrelevant information, including the distractor (Scalf et al. 2013). Such biased competition processes might explain the nonlinear ERP effects for perceptual load. Here, top-down control is exerted to solve the task and shield target detection from emotional distracters, but such top-down inhibitory processes collapse at the highest perceptual difficulty level.

Finally, we could validate the expected main effects of load, leading to linearly decreasing behavioral performance as well as linearly decreased EPN and LPP amplitudes. This is in line with numerous studies showing that higher task difficulty often results in longer reaction times and reduces late amplitudes, explained by a smearing of effects due to more variable decision points or a higher response variability (e.g., see O’Connell et al. 2012; Twomey et al. 2015).

### Constraints on Generality

With regard to our study’s findings, there are some constraints that have to be mentioned. As noted above, systematic reaction time and ERP differences are observed by the linear increase in perceptual load difficulty. This also led to a higher number of error trials, which might have affected late ERPs. We therefore calculated ERPs analyses restricted to correct trials only, showing that similar interactions were found for the N1, EPN, and LPP (see Supplementary Materials). Furthermore, we realized a short presentation duration of the load task to avoid systematic eye movements. Studies examining visual attention rely often on full eye movement rejection to avoid condition differences in number or intensity of blinks or saccades (as for example recommended by Luck (2014)). A further point needing discussion is related to differences in examined EOG activity, showing for vertical eye movements a main effect in the LPP range (see Fig. 5). While no interactions were found, this difference in eye movements across load conditions could indicate that distractor pictures falls on different locations of the visual field and thus receives different amounts of attention. This could have differentially influenced the strength of the emotion effect in the different load conditions. Finally, pictures were not fully matched regarding brightness, which can modulate early ERPs (De Cesarei et al. 2017, see also the Supplementary Materials). We cannot exclude complex interactions of brightness and load levels for the emotion-neutral differences. However, in comparison with a high-powered study using the same pictures without a load task (Schindler and Straube 2020), we see a tremendously different pattern regarding the negative-neutral and positive-neutral differences for the P1, N1, EPN, and LPP.

## Conclusion

To conclude, we detected a systematic nonlinear influence of linearly increasing perceptual load on the emotional modulation of ERP components, including the N1, EPN, and LPP. Importantly, ERP differences between emotional and neutral pictures reversed at load levels 2–4, being maximal at load level 3. These results indicate that emotional ERP modulations are not simply absent, but are actively suppressed already during early processing stages under conditions of higher load. However, they also show that these processes may collapse at a certain point of load difficulty.

## Notes

We thank Nele Johanna Bögemann for her corrections, and all participants contributing to this study. *Conflict of Interest*: None declared.

## Supplementary Material

M081_Supplementary_materials_tgaa040
