# Supplementary material for: Nonlinear Effects of Linearly Increasing Perceptual Load on ERPs to Emotional Pictures
Source: Cereb Cortex Commun. 2020 Jul 29;1(1):tgaa040. doi: 10.1093/texcom/tgaa040 (PMC8153054; doi:10.1093/texcom/tgaa040)
Supplement: M081_Supplementary_materials_tgaa040 [file m081_supplementary_materials_tgaa040.docx]

**Nonlinear effects of linearly increasing perceptual load on ERPs to emotional pictures**

**-**

SUPPLEMENTARY MATERIAL

Sebastian Schindler^1,2*^, Laura Gutewort^1^, Maximilian Bruchmann^1,2^, Robert Moeck^1^, Thomas Straube^1,2^

^1^ Institute of Medical Psychology and Systems Neuroscience, University of Muenster

^2^ Otto Creutzfeldt Center for Cognitive and Behavioral Neuroscience, University of Muenster

* Corresponding author

**Correspondence address**

Institute of Medical Psychology and Systems Neuroscience

University of Münster

Von-Esmarch-Str. 52, D-48149 Münster, Germany

e-mail: [sebastian.schindler@ukmuenster.de](mailto:sebastian.schindler@ukmuenster.de)

1. **Image statistics**

We compared statistics for brightness and frequency information between the image sets (Torralba and Oliva 2003; Bainbridge and Oliva 2015). Firstly, regarding picture brightness, we observed that negative pictures exhibited the lowest average grey levels. These, however, differed only from positive images (see Supplementary Figure 1). Here, a Bayesian ANOVA did not find evidence for a difference (BF_M_ = 0.721). However, post-hoc Bayesian *t*-tests showed that there was evidence for a difference between negative and positive images (BF_10, U_ = 3.28), with brighter positive images. No differences were found for negative compared to neutral (BF_10, U_ = 0.60), or positive compared to neutral images (BF_10, U_ = 0.25).


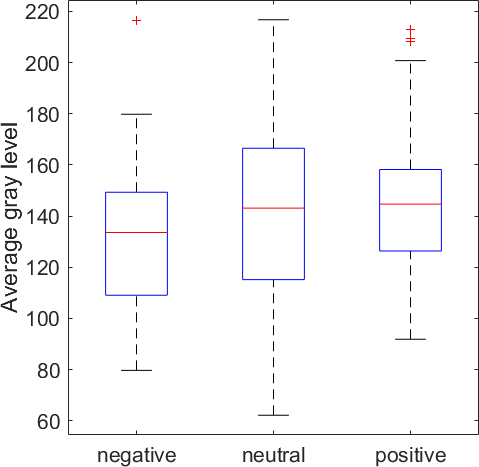


**Supplementary Figure S1:** Image statistics for the average picture brightness indicated by grey level values for negative, neutral, and positive pictures.

Regarding spatial frequencies, we found that bootstrap confidence intervals overlapped between negative, neutral and positive images in lower frequencies. From 30 cycles onwards, confidence intervals between positive and negative pictures did not overlap, indicating a higher power of positive pictures in these higher frequencies (see Supplementary Figure 2). Positive images the highest relative power in all color spectra, followed by neutral and lastly by negative images. Furthermore, neutral pictures elicited distinct peaks around 44 and 98 Hz. Here, a fine-grained check revealed that these peaks (in the neutral condition) were caused by one single neutral image with strong regularities in horizontal and vertical lines (IAPS tech report image number 7185, ‘abstract art’). The detailed ratings per image (taken from the 2008 tech report) as well as information on all used pictures can be retrieved from the OSF project (https://osf.io/8rs7b/), which is linked to the registered study (https://osf.io/qn38r).


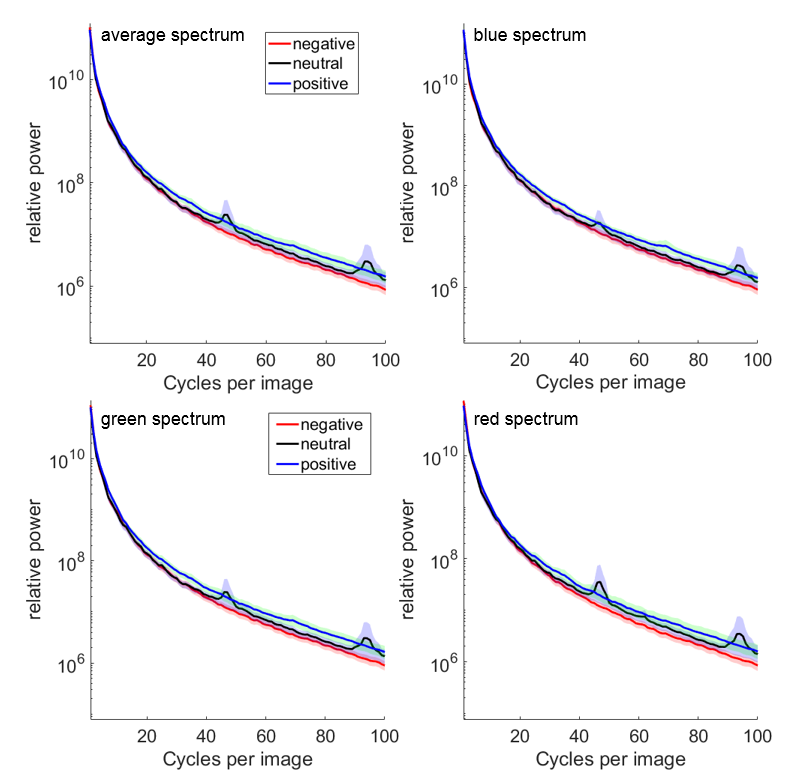
**Supplementary Figure S2:** Image statistics for the blue, green, and red spectra. Displayed is the relative power across spatial frequency bands. Bootstrap confidence intervals around each line are highlighted by green, blue, and red colors.

1. **Limitation of the P1 main emotion effect**

For the main emotion effect on the P1 amplitude, it is important to note that such early effects are – at least partly – modulated by low-level properties (De Cesarei et al., 2017). Even though we did not observe a higher power for negative pictures in low spatial frequencies (found to modulate ERPs in the P1 to P2 range, see De Cesarei et al. 2013), we observed a relatively higher power for positive pictures in higher frequencies (see Supplementary Figure S2). Such frequency differences might have influenced picture processing (De Cesarei and Codispoti 2011, 2012). Regarding brightness, post-hoc tests showed differences between negative and positive pictures, which could have partly biased a stronger P1 response to the negative pictures.

1. **ERP analyses restricted to correct trials only**

As EEG data included trials with incorrect responses, these might have affected the ERP modulations, especially late ERP effects. To control for difficulty effects on our observed interactions, we additionally calculated ERPs only for correct trials. EEG pre-processing and analyses were unchanged (for details about pre-processing and used sensors and intervals, see the methods paragraphs in the main document). Given the linear accuracy decrease for increasing load, kept trial numbers were unequal: On average, 38 trials (~63 percent) were kept for averaging. On average, for load level 1, 51 trials were kept, for level 2, 43 trials, for level 3, 35 trials and for level 4, only 23 trials. There were no differences in kept trials between emotion (*F*_(2,58)_ = 0.92, *p =* .404, partial η² = .031), but strong main effects of load (*F*_(2.21,64.17)_ = 299.09, *p <* .001, partial η² = .912), and no interaction of load and emotion (*F*_(6,174)_ = 0.67, *p =* .675, partial η² = .023). For the main effect of load, analyses show linear decreasing number of kept trials with increasing load (*F*_(1,29)_ = 889.66, *p >* .001, partial η² = .968; 100% variance explained).

**P1 (80 – 100 ms)**

Regarding the P1, a main effect of emotion was found (*F*_(2,58)_ = 3.99, *p =* .024, partial η² = .121; see Supplementary Figure S3), but no main effect of perceptual load was detected (*F*_(3,87)_ = 0.22, *p =* .880, partial η² = .008). With respect to the main effect of emotion, negative pictures elicited a larger P1 amplitude when compared to positive (*p =* .018), but not when compared to neutral pictures (*p =* .137), the latter two not differing (*p =* .140). There was no significant interaction of emotion and load (*F*_(4.31,124.99)_ = 0.57, *p =* .698, partial η² = .019).

**N1 (110 – 170 ms)**

For the N1, a main effect of emotion (*F*_(2,58)_ = 13.55, *p <* .001, partial η² = .318), but no main effect of perceptual load was found (*F*_(3,87)_ = 0.86, *p =* .467, partial η² = .029). Regarding the main effect of emotion, negative pictures elicited a smaller N1 amplitude compared to both neutral (*p <* .001) and positive pictures (*p =* .002). Compared to neutral pictures, positive pictures also elicited a smaller N1 amplitude (*p =* .044).

There was a significant interaction of emotion and load (*F*_(4.15,120.26)_ = 2.75, *p =* .030, partial η² = .087; see Supplementary Figure S3). Polynomial trends were computed for amplitude differences between negative and neutral pictures, showing a significant linear (*F*_(1,29)_ = 4.49, *p =* .043, partial η² = .134; 66% variance explained), and cubic trend (*F*_(1,29)_ = 4.66 *p =* .039, partial η² = .138; 18% variance explained), but no significant quadratic trend (*F*_(1,29)_ = 2.63, *p =* .116, partial η² = .083; 16% variance explained). When examining such differences between positive and neutral pictures, there were no significant linear (*F*_(1,29)_ = 1.19, *p =* .284, partial η² = .039; 15% variance explained) or quadratic contrasts either (*F*_(1,29)_ = 3.02, *p =* .093, partial η² = .094; 26% variance explained). However, a cubic trend reached significance (*F*_(1,29)_ = 6.85, *p =* .014, partial η² = .191; 59% variance explained).

**
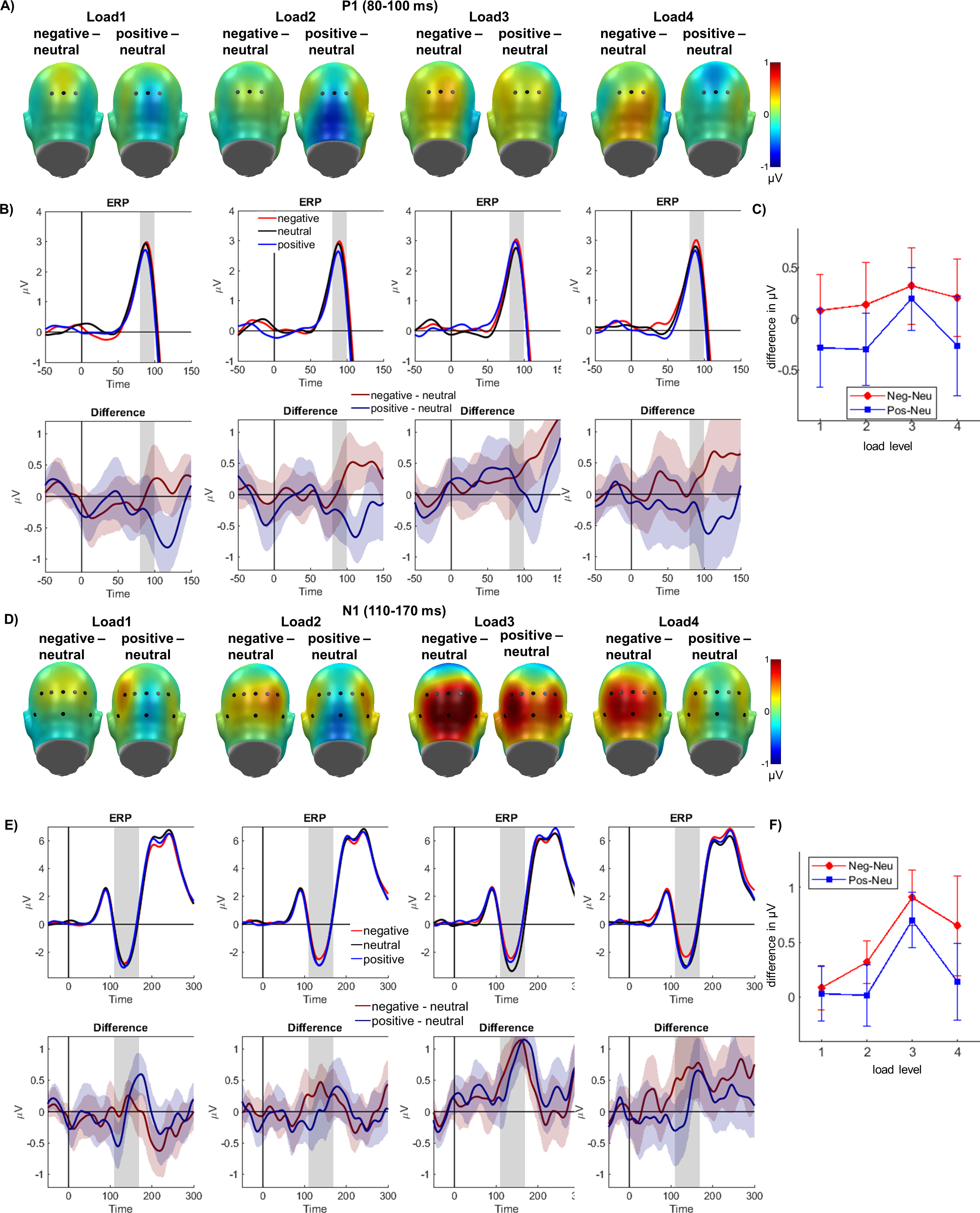
Supplementary Figure S3. P1 (A-C) and N1 (D-F) effects for correct trials only.** **A and D)** Difference topographies between negative and neutral and between positive and neutral pictures for each load level, highlighting the electrodes for the P1 (A) and N1 (D) ROI. **B and E)** Averages for all emotion conditions over the P1 (B) and N1 (E) electrode clusters and their difference plots (negative-neutral and positive-neutral), displayed separately for each load level. **C and F)** Average of differences (negative-neutral and positive-neutral) over the entire P1 (C) and N1 (F) time window for all load levels. Error bars depict 95% CIs.

**EPN**

With respect to the EPN, both main effects of emotion (*F*_(1.62,46.94)_ = 7.59, *p* = .003, partial η² = .207) and perceptual load reached significance (*F*_(3,87)_ = 7.12, *p <* .001, partial η² = .197). Regarding the main effect of emotion, neutral pictures elicited a larger EPN than negative (*p =* .002) and positive stimuli (*p =* .008). The latter negative and positive conditions did not exhibit significantly different amplitude values (*p =* .098). For the main effect of load, polynomial trends showed a linear decrease in EPN amplitude values with increasing load (*F*_(1,29)_ = 12.47, *p* = .001, partial η² = .301 ; 91% variance explained). In addition, there was a significant interaction effect of emotion and load (*F*_(6,174)_ = 2.51, *p =* .024, partial η² = .080; see Supplementary Figure S4). Here, polynomial trends computed for differences between negative and neutral pictures showed a significant linear trend (*F*_(1,29)_ = 5.30, *p =* .029, partial η² = .155; 95% variance explained), but no significant quadratic (*F*_(1,29)_ = 0.50, *p =* .488, partial η² = .017; 4% variance explained), or cubic contrast (*F*_(1,29)_ = 0.07, *p =* .800, partial η² = .002; 1% variance explained). Regarding the differences between positive and neutral pictures, there was no significant linear (*F*_(1,29)_ = 0.17, *p =* .682, partial η² = .006; 3% variance explained) or quadratic trend (*F*_(1,29)_ = 1.28, *p =* .268, partial η² = .042; 19% variance explained), but a cubic contrast reached significance (*F*_(1,29)_ = 5.02, *p =* .033, partial η² = .148; 78% variance explained).

**LPP**

Regarding the LPP, no significant main effect of emotion was found (*F*_(2,58)_ = 0.63, *p =* .537, partial η² = .021), but a main effect of perceptual load was detected (*F*_(2.16,62.59)_ = 16.61, *p <* .001, partial η² = .364). Polynomial trends showed a significant linear trend of decreasing LPP amplitudes with increasing load (*F*_(1,29)_ = 27.07, *p* < .001, partial η² = .483; 99% variance explained).


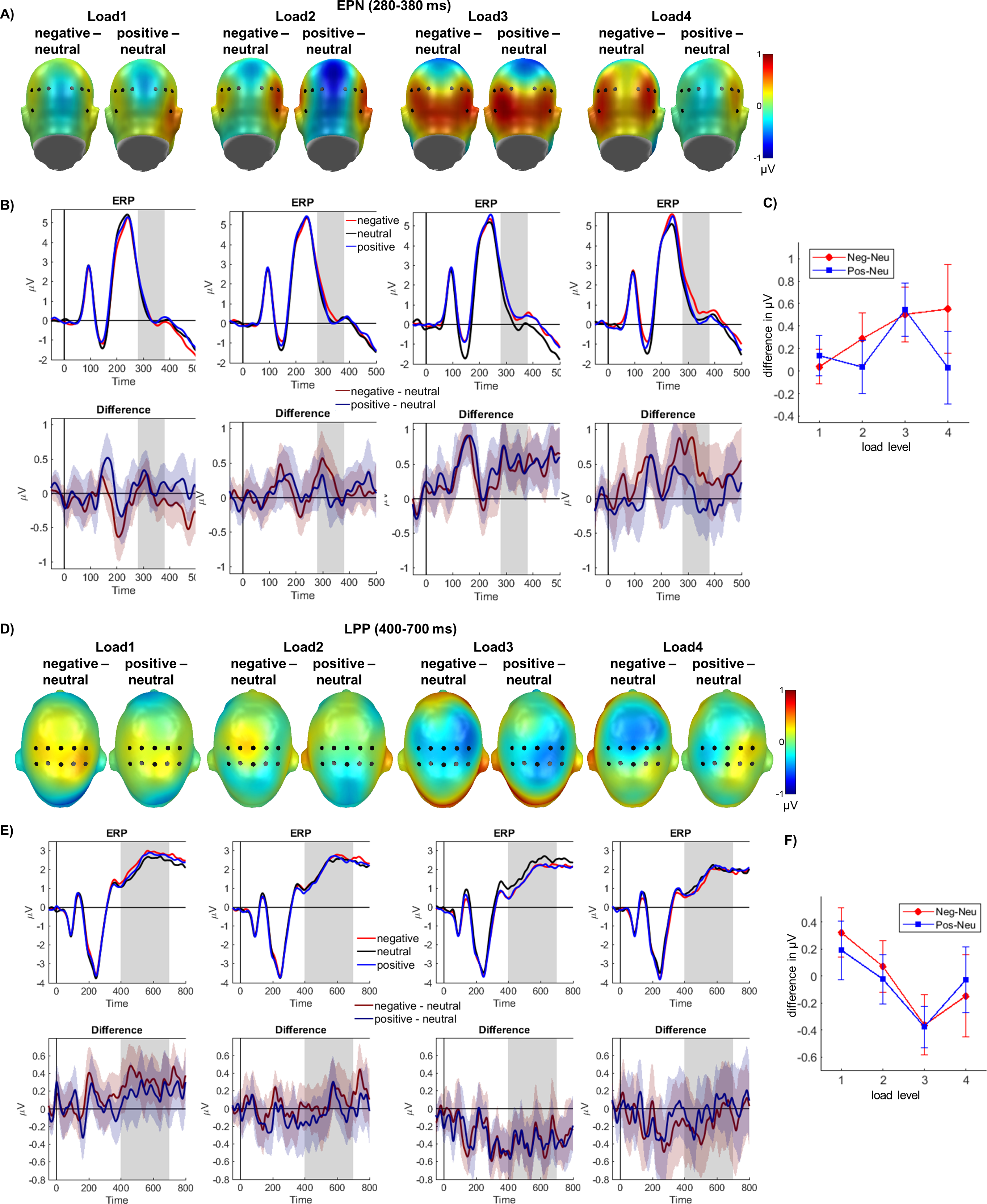


**Supplementary Figure S4. EPN (A-C) and LPP (D-F) effects for correct trials only. A and D)** Difference topographies between negative and neutral and between positive and neutral pictures for each load level, highlighting the electrodes for the EPN (A) and LPP (C) ROI. **B and E)**Averages for all emotion conditions over the P1 (B) and N1 (E) electrode clusters and their difference plots (negative-neutral and positive-neutral), displayed separately for each load level. **C and F)** Average of differences (negative-neutral and positive-neutral) over the entire EPN (C) and LPP (F) time window for all load levels. Error bars depict 95% CIs.

Furthermore, there was a significant interaction of emotion and load (*F*_(4.15,120.42)_ = 2.53, *p =* .042, partial η² = .080; see Supplementary Figure S4). Polynomial trends computed for the differences between negative and neutral pictures showed a significant linear (*F*_(1,29)_ = 5.66, *p =* .0024, partial η² = .163; 65% variance explained), but no significant cubic contrast (*F*_(1,29)_ = 2.13, *p =* .155, partial η² = .068; 22% variance explained), or quadratic trend (*F*_(1,29)_ = 2.15, *p =* .153, partial η² = .069; 13% variance explained). With regard to the differences between positive and neutral pictures, there was no significant linear (*F*_(1,29)_ = 1.91, *p =* .177, partial η² = .062; 27% variance explained), or cubic trend (*F*_(1,29)_ = 2.85, *p =* .102, partial η² = .090; 18% variance explained), but a significant quadratic trend (*F*_(1,29)_ = 6.20, *p =* .019, partial η² = .177; 55% variance explained).

**Supplementary File References**

Bainbridge WA, Oliva A. 2015. A toolbox and sample object perception data for equalization of natural images. Data Brief. 5:846–851.

De Cesarei A, Codispoti M. 2011. Scene identification and emotional response: which spatial frequencies are critical? J Neurosci. 31:17052–17057.

De Cesarei A, Codispoti M. 2012. Spatial frequencies and emotional perception. Rev Neurosci. 24:89–104.

De Cesarei A, Loftus GR, Mastria S, Codispoti M. 2017. Understanding natural scenes: Contributions of image statistics. Neurosci Biobehav Rev. 74:44–57.

De Cesarei A, Mastria S, Codispoti M. 2013. Early Spatial Frequency Processing of Natural Images: An ERP Study. PLOS ONE. 8:e65103.

Torralba A, Oliva A. 2003. Statistics of natural image categories. Netw Comput Neural Syst. 14:391–412.
